# Supplementary material for: A national cohort study of long-term opioid prescription and sociodemographic and health care-related risk factors
Source: Commun Med (Lond). 2025 Sep 17;5:390. doi: 10.1038/s43856-025-01135-8 (PMC12443973; doi:10.1038/s43856-025-01135-8)
Supplement: Supplementary file 2 — Supplementary Information [file 43856_2025_1135_MOESM2_ESM.pdf]

Supplementary Information

Article title: A national cohort study of long-term opioid prescription and sociodemographic and health care-related risk factors

Authors: Cecilia Krüger, Johan Franck, Härje Widing, Jonas Hällgren, Mika Gissler, Jeanette Westman

Supplementary Figure 1. Odds ratios (ORs) and 95% confidence intervals (CIs) for long-term opioid use (>3 months) in a sensitivity analysis where the permissible gap between dispensing dates was reduced from 180 days to 120 days (reference: short-term use defined as ≤3 months)

Characteristics	Reference	OR (95% CI)
**Age group**		
35-49	18-34	1.27 (1.24-1.31)
50-64	18-34	1.59 (1.55-1.64)
**Sex**		
Female	Male	0.94 (0.92-0.96)
**Cohabitation status**		
Cohabiting	Not cohabiting	0.88 (0.86-0.90)
**Level of education**		
Primary	Tertiary	1.57 (1.52-1.62)
Secondary	Tertiary	1.31 (1.28-1.34)
**Country of birth**		
Other European	Sweden	1.01 (0.97-1.05)
Other	Sweden	1.03 (1.00-1.07)
**Size of municipality of residence**		
Medium-sized towns	Large cities	1.22 (1.19-1.25)
Small towns/rural areas	Large cities	1.23 (1.20-1.27)
**Cancer**		
Yes	No	2.39 (2.29-2.50)
**Recent external injury**		
Yes	No	0.76 (0.74-0.78)
**Substance use disorders**		
Yes	No	1.55 (1.48-1.62)
**Number of psycholeptic drug classes**		
1	0	1.63 (1.59-1.67)
2	0	2.11 (2.04-2.17)
3	0	2.77 (2.62-2.93)
**Prescriber care level**		
Primary care	Specialized care	2.15 (2.10-2.19)

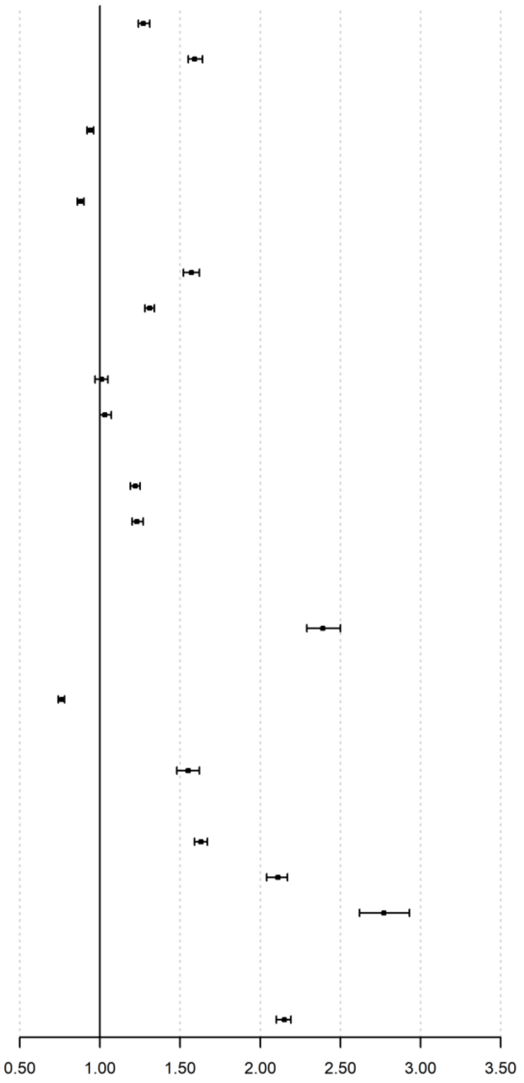

Abbreviations: CI, confidence interval; OR, odds ratio

n=754 982 opioid-naïve individuals included in the analysis.

Health care related characteristics are based on diagnoses in period prior to initial prescription: 1-year history of cancer, 3-month history of an external injury, and 5-year history of substance use disorders and number of psycholeptic medications. History of prescription psycholeptic drug use is a proxy for number of common psychiatric disorders.

Supplementary Table 1. Odds ratios (ORs) and 95% confidence intervals (CIs) for the duration of long-term opioid use by user characteristics in a sensitivity analysis where the permissible gap between dispensing dates was reduced from 180 days to 120 days (reference category: short-term use, defined as ≤ 3 months)

Characteristics	Reference	>3-6 months n=23 112 OR (95% CI)	>6-12 months n=8 139 OR (95% CI)	>12 months n=7 082 OR (95% CI)
**Sociodemographic characteristics**				
**Age group**				
35-49	18-34	1.20 (1.16-1.25)	1.34 (1.26-1.43)	1.50 (1.39-1.62)
50-64	18-34	1.44 (1.39-1.49)	1.73 (1.63-1.84)	2.08 (1.94-2.22)
**Sex**				
Female	Male	0.97 (0.95-1.00)	0.93 (0.89-0.97)	0.86 (0.82-0.90)
**Cohabitation status**				
Cohabitating	Not cohabiting	0.93 (0.90-0.95)	0.87 (0.83-0.91)	0.74 (0.71-0.78)
**Level of education**				
Primary	Tertiary	1.35 (1.29-1.40)	1.72 (1.61-1.84)	2.33 (2.16-2.50)
Secondary	Tertiary	1.22 (1.18-1.25)	1.39 (1.32-1.47)	1.62 (1.53-1.72)
**Country of birth**				
Other European	Sweden	1.05 (1.00-1.1)	0.99 (0.91-1.06)	0.93 (0.85-1.01)
Other	Sweden	1.09 (1.05-1.14)	1.06 (0.99-1.14)	0.81 (0.74-0.89)
**Size of municipality of**				
Medium-sized towns	Large cities	1.07 (1.04-1.11)	1.4 (1.33-1.48)	1.67 (1.58-1.77)
Smaller towns/rural areas	Large cities	1.08 (1.04-1.12)	1.38 (1.30-1.47)	1.74 (1.63-1.86)
**Health care-related characteristics [a]**				
**Cancer**				
Yes	No	2.20 (2.08-2.33)	2.37 (2.16-2.6)	3.23 (2.95-3.54)
**Recent external injury**				
Yes	No	0.84 (0.81-0.87)	0.69 (0.65-0.74)	0.56 (0.52-0.61)
**Substance use disorders**				
Yes	No	1.39 (1.31-1.48)	1.63 (1.49-1.79)	1.89 (1.73-2.06)
**Number of psycholeptic medication classes [b]**				
1	0	1.44 (1.39-1.48)	1.76 (1.67-1.86)	2.34 (2.21-2.48)
2	0	1.69 (1.62-1.76)	2.34 (2.20-2.50)	3.65 (3.42-3.90)
3	0	2.02 (1.87-2.19)	3.07 (2.74-3.44)	5.61 (5.06-6.21)
**Prescriber level**				
Primary care	Specialized	1.82 (1.77-1.87)	2.56 (2.44-2.68)	3.08 (2.93-3.24)

Abbreviations: CI, confidence interval; OR, odds ratio

n=754 982 opioid-naïve individuals included in the analysis.

[a] Based on diagnoses in period prior to initial prescription: 1-year history of cancer, 3-month history of an external injury, and 5-year history of substance use disorders and number of psycholeptic medications (Supplementary Table 1).
[b] Individual-level, 5-year history of prescription psycholeptic drug use is a proxy for number of common psychiatric disorders.

Supplementary Figure 2. Odds ratios (ORs) and 95% confidence intervals (CIs) for long-term opioid use (>3 months) in a sensitivity analysis where the permissible gap between dispensing dates was reduced to 90 days and additionally required that at least 2 prescriptions were dispensed within days 0-90, and at least one more during days 91-180. (reference: short-term use defined as ≤3 months)

Characteristics	Reference	OR (95% CI)
**Age group**		
35-49	18-34	1.40 (1.34-1.45)
50-64	18-34	1.82 (1.75-1.89)
**Sex**		
Female	Male	0.89 (0.86-0.91)
**Cohabitation status**		
Cohabiting	Not cohabiting	0.84 (0.82-0.87)
**Level of education**		
Primary	Tertiary	1.68 (1.61-1.75)
Secondary	Tertiary	1.35 (1.31-1.40)
**Country of birth**		
Other European	Sweden	0.94 (0.90-0.99)
Other	Sweden	0.94 (0.90-0.99)
**Size of municipality of residence**		
Medium-sized towns	Large cities	1.35 (1.31-1.39)
Small towns/rural areas	Large cities	1.38 (1.33-1.43)
**Cancer**		
Yes	No	2.88 (2.73-3.04)
**Recent external injury**		
Yes	No	0.77 (0.74-0.81)
**Substance use disorders**		
Yes	No	1.70 (1.61-1.80)
**Number of psycholeptic drug classes**		
1	0	1.76 (1.70-1.82)
2	0	2.35 (2.26-2.45)
3	0	3.34 (3.12-3.57)
**Prescriber care level**		
Primary care	Specialized care	2.53 (2.46-2.60)

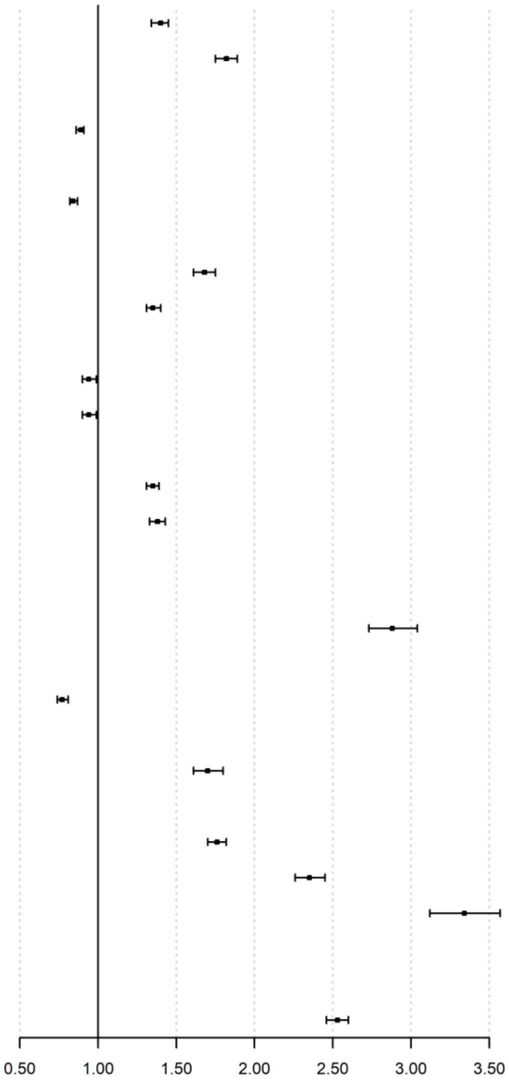

Abbreviations: CI, confidence interval; OR, odds ratio

n=754 982 opioid-naïve individuals included in the analysis.

Health care related characteristics are based on diagnoses in period prior to initial prescription: 1-year history of cancer, 3-month history of an external injury, and 5-year history of substance use disorders and number of psycholeptic medications. History of prescription psycholeptic drug use is a proxy for number of common psychiatric disorders.

Supplementary Table 2. Odds ratios (ORs) and 95% confidence intervals (CIs) for the duration of long-term opioid use by user characteristics in a sensitivity analysis where the permissible gap between dispensing dates was reduced to 90 days and additionally required that at least 2 prescriptions were dispensed within days 0-90, and at least one more during days 91-180. (reference: short-term use defined as ≤3 months)

Characteristics	Reference	**>3-6 months n=23 112** OR (95% CI)	**>6-12 months n=8 139** OR (95% CI)	**>12 months n=7 082** OR (95% CI)
**Sociodemographic characteristics**				
**Age group**				
35-49	18-34	1.36 (1.29-1.43)	1.35 (1.24-1.46)	1.6 (1.46-1.75)
50-64	18-34	1.71 (1.63-1.79)	1.82 (1.69-1.96)	2.19 (2.02-2.38)
**Sex**				
Female	Male	0.90 (0.87-0.94)	0.89 (0.84-0.94)	0.83 (0.78-0.88)
**Cohabitation status**				
Cohabitating	Not cohabiting	0.88 (0.85-0.91)	0.85 (0.80-0.90)	0.75 (0.71-0.80)
**Level of education**				
Primary	Tertiary	1.43 (1.35-1.51)	1.8 (1.66-1.96)	2.41 (2.21-2.64)
Secondary	Tertiary	1.25 (1.20-1.30)	1.42 (1.33-1.52)	1.66 (1.54-1.79)
**Country of birth**				
Other European	Sweden	1.01 (0.95-1.08)	0.88 (0.80-0.97)	0.83 (0.75-0.92)
Other	Sweden	1.04 (0.98-1.10)	0.93 (0.85-1.02)	0.71 (0.64-0.80)
**Size of municipality of residence**				
Medium-sized towns	Large cities	1.20 (1.15-1.25)	1.48 (1.39-1.58)	1.74 (1.62-1.87)
Smaller towns/rural areas	Large cities	1.23 (1.18-1.29)	1.46 (1.36-1.57)	1.77 (1.64-1.92)
**Health care-related characteristics [a]**				
**Cancer**				
Yes	No	2.68 (2.49-2.87)	2.71 (2.43-3.02)	3.74 (3.36-4.15)
**Recent external injury**				
Yes	No	0.88 (0.84-0.93)	0.69 (0.64-0.75)	0.58 (0.53-0.64)
**Substance use disorders**				
Yes	No	1.57 (1.45-1.69)	1.69 (1.52-1.89)	2.00 (1.81-2.21)
**Number of psycholeptic medication classes [b]**				
1	0	1.54 (1.48-1.61)	1.87 (1.75-1.99)	2.42 (2.25-2.60)
2	0	1.91 (1.81-2.01)	2.57 (2.38-2.77)	3.69 (3.41-3.99)
3	0	2.42 (2.2-2.67)	3.58 (3.15-4.08)	6.27 (5.56-7.06)
**Prescriber level**				
Primary care	Specialized	2.26 (2.18-2.35)	2.78 (2.63-2.94)	3.10 (2.91-3.30)

Abbreviations: CI, confidence interval; OR, odds ratio

n=754 982 opioid-naïve individuals included in the analysis.

[a] Based on diagnoses in period prior to initial prescription: 1-year history of cancer, 3-month history of an external injury, and 5-year history of substance use disorders and number of psycholeptic medications (Supplementary Table 1).
[b] Individual-level, 5-year history of prescription psycholeptic drug use is a proxy for number of common psychiatric disorders.
