# Supplementary material for: A national cohort study of long-term opioid prescription and sociodemographic and health care-related risk factors
Source: Commun Med (Lond). 2025 Sep 17;5:390. doi: 10.1038/s43856-025-01135-8 (PMC12443973; doi:10.1038/s43856-025-01135-8)
Supplement: Supplementary file 3 — Description of Additional Supplementary Files [file 43856_2025_1135_MOESM3_ESM.pdf]

Description of Additional Supplementary Files

File name- Supplementary Data 1

File description - Table containing definitions of variables used in the study and data sources for each variable.

File name- Supplementary Data 2

File description – Table containing formulation characteristics and morphine milligram equivalent (MME) conversion factors for opioid analgesic formulations included in the study.
