# Supplementary material for: A national cohort study of long-term opioid prescription and sociodemographic and health care-related risk factors
Source: Commun Med (Lond). 2025 Sep 17;5:390. doi: 10.1038/s43856-025-01135-8 (PMC12443973; doi:10.1038/s43856-025-01135-8)
Supplement: Supplementary file 6 — Reporting summary [file 43856_2025_1135_MOESM6_ESM.pdf]

nature portfolio

Corresponding author(s): Cecilia Krüger

Last updated by author(s): Sep 3, 2025

Reporting Summary

Nature Portfolio wishes to improve the reproducibility of the work that we publish. This form provides structure for consistency and transparency in reporting. For further information on Nature Portfolio policies, see our Editorial Policies and the Editorial Policy Checklist.

Statistics

For all statistical analyses, confirm that the following items are present in the figure legend, table legend, main text, or Methods section.

n/a	Confirmed	
☐	☒	The exact sample size (*n*) for each experimental group/condition, given as a discrete number and unit of measurement
☐	☒	A statement on whether measurements were taken from distinct samples or whether the same sample was measured repeatedly
☐	☒	The statistical test(s) used AND whether they are one- or two-sided<br>*Only common tests should be described solely by name; describe more complex techniques in the Methods section.*
☐	☒	A description of all covariates tested
☐	☒	A description of any assumptions or corrections, such as tests of normality and adjustment for multiple comparisons
☐	☒	A full description of the statistical parameters including central tendency (e.g. means) or other basic estimates (e.g. regression coefficient) AND variation (e.g. standard deviation) or associated estimates of uncertainty (e.g. confidence intervals)
☒	☐	For null hypothesis testing, the test statistic (e.g. *F*, *t*, *r*) with confidence intervals, effect sizes, degrees of freedom and *P* value noted<br>*Give P values as exact values whenever suitable.*
☒	☐	For Bayesian analysis, information on the choice of priors and Markov chain Monte Carlo settings
☒	☐	For hierarchical and complex designs, identification of the appropriate level for tests and full reporting of outcomes
☒	☐	Estimates of effect sizes (e.g. Cohen's *d*, Pearson's *r*), indicating how they were calculated

Our web collection on statistics for biologists contains articles on many of the points above.

Software and code

Policy information about availability of computer code

Data collection	No software was used for data collection (i.e. register-based data).
Data analysis	Data was analyzed using SAS statistical software version 9.4. The code used to generate the results in this study is available at Zenodo under DOI: 10.5281/zenodo.17038451.

For manuscripts utilizing custom algorithms or software that are central to the research but not yet described in published literature, software must be made available to editors and reviewers. We strongly encourage code deposition in a community repository (e.g. GitHub). See the Nature Portfolio guidelines for submitting code & software for further information.

Data

Policy information about availability of data

All manuscripts must include a data availability statement. This statement should provide the following information, where applicable:

- Accession codes, unique identifiers, or web links for publicly available datasets
- A description of any restrictions on data availability
- For clinical datasets or third party data, please ensure that the statement adheres to our policy

The dataset provided to us after application to the register holders cannot be shared due to data protection and privacy regulations. However, researchers can get access to data for all variables in the dataset by request to National Board of Health and Welfare and Statistics Sweden.

Research involving human participants, their data, or biological material

Policy information about studies with human participants or human data. See also policy information about sex, gender (identity/presentation), and sexual orientation and race, ethnicity and racism.

Reporting on sex and gender	Sex (female/male) is reported based on the legal sex as registered in the Total Population Register. Gender is not included. Sex is included in the analysis as a covariate as males and females may have different prescription rates for opioids. However, sex-specific reporting was not considered a key question in this study as there were no major differences found in this data set.
Reporting on race, ethnicity, or other socially relevant groupings	No variables relating to race or ethnicity were included. We do report country of birth classified in three categories based on administrative data in the Total Population Register: Sweden, European country other than Sweden (abbr. other European), or outside Europe or missing data on country of birth (abbr. other). Country of birth is important to include in the analysis as healthcare uptake may differ based on country of birth, which can be a proxy for socicutural differences in medication use and health outcomes.
Population characteristics	Assessed sociodemographic population characteristics include: Age, legal sex, cohabitation status, level of education, country of birth, size of municipality of residence. Health care-related characteristics include: history of diagnoses related to cancer, external injury, substance use disorders, other mental health disorders, number and types of psycholeptic drug classes prescribed, and characterstics related to their opioid prescription(s).
Recruitment	This is a national cohort study based on register data (i.e., no patients were directly contacted or recruited).<br>See eligibility criteria below.
Ethics oversight	The study was approved by the national Swedish Ethical Review Authority under Dnr. 2019-00516

Note that full information on the approval of the study protocol must also be provided in the manuscript.

Field-specific reporting

Please select the one below that is the best fit for your research. If you are not sure, read the appropriate sections before making your selection.

☐ Life sciences ☒ Behavioural & social sciences ☐ Ecological, evolutionary & environmental sciences

For a reference copy of the document with all sections, see nature.com/documents/nr-reporting-summary-flat.pdf

Behavioural & social sciences study design

All studies must disclose on these points even when the disclosure is negative.

Study description	Quantitative study. This is a nation-wide observational cohort study using Swedish register data.
Research sample	The sample was selected from Swedish nationwide registers. The population meets the inclusion criteria of residing in Sweden during the study period. These data provide an opportunity to assess risk factors and outcomes on the population level by linking several different registers.<br>The specific sample in this study is cohort of Swedish residents aged 18 through 64 years who received an initial prescription of an opioid analgesic, classified under Anatomical Therapeutic Chemical (ATC) code N02A, during the observational period of 1 January, 2016 through 31st December, 2020 while previously being 5-year opioid-naive (see definition below).
Sampling strategy	All individuals in the registers who met the pre-established inclusion and exclusion criteria were included in the study population. As this is a comprehensive sample from the register, no sample size calculation was performed.<br>Criteria for inclusion were: registered as living in Sweden in the Total population register during the study period (2016 to 2020), aged 18 through 64 years, and received an initial prescription of an opioid during the study period. To fulfill the criteria of being opioid-naive, individuals must not have had prescription opioid use, opioid use disorder, or recieved opioid maintenence treatment in the 5 years prior to the initial prescription. N=391 277 were excluded as non-naive.
Data collection	The population was identified in the Swedish national registers, and was exported to an analysis dataset.
Timing	The observational period was 1 January, 2016 through 31st December, 2020.
Data exclusions	Individuals were excluded from the cohort died (n=9 206) or emigrated (n=3 186) during follow-up as it would not be possible to follow their outcomes. As common in other studies, individuals dispensed more than 4000 morphine milligram equivalents in the initiation month were excluded (917). Such doses are too high for opioid-naive patients (overdose and respiratory depression are risks at this level) and indicates that such patients may not be naive. Individuals who were missing data on prescriber level were excluded as this is an important covariate in the regression analysis (380). These criteria were pre-established (see study flowchart, Figure 1).
Non-participation	Not relevant as this is a nationwide register-based study.

Randomization	Not relevant as this is an observational study.

Reporting for specific materials, systems and methods

We require information from authors about some types of materials, experimental systems and methods used in many studies. Here, indicate whether each material, system or method listed is relevant to your study. If you are not sure if a list item applies to your research, read the appropriate section before selecting a response.

Materials & experimental systems

n/a	Involved in the study
☒	☐ Antibodies
☒	☐ Eukaryotic cell lines
☒	☐ Palaeontology and archaeology
☒	☐ Animals and other organisms
☒	☐ Clinical data
☒	☐ Dual use research of concern
☒	☐ Plants

Methods

n/a	Involved in the study
☒	☐ ChIP-seq
☒	☐ Flow cytometry
☒	☐ MRI-based neuroimaging

Plants

Seed stocks	*Report on the source of all seed stocks or other plant material used. If applicable, state the seed stock centre and catalogue number. If plant specimens were collected from the field, describe the collection location, date and sampling procedures.*
Novel plant genotypes	*Describe the methods by which all novel plant genotypes were produced. This includes those generated by transgenic approaches, gene editing, chemical/radiation-based mutagenesis and hybridization. For transgenic lines, describe the transformation method, the number of independent lines analyzed and the generation upon which experiments were performed. For gene-edited lines, describe the editor used, the endogenous sequence targeted for editing, the targeting guide RNA sequence (if applicable) and how the editor was applied.*
Authentication	*Describe any authentication procedures for each seed stock used or novel genotype generated. Describe any experiments used to assess the effect of a mutation and, where applicable, how potential secondary effects (e.g. second site T-DNA insertions, mosiacism, off-target gene editing) were examined.*
